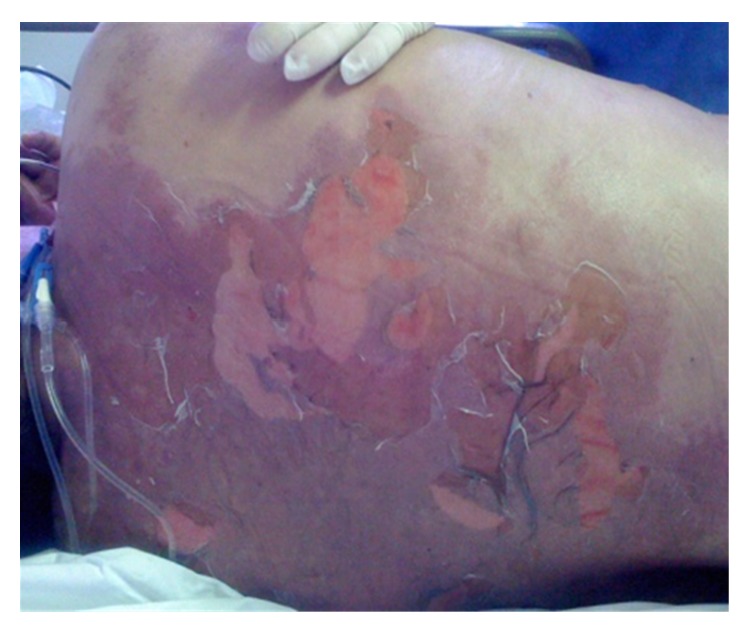# Cephazolin-Induced Toxic Epidermal Necrolysis Treated with Intravenous Immunoglobulin and N-Acetylcysteine

**DOI:** 10.1155/2012/931528

**Published:** 2012-04-19

**Authors:** Carlos Saavedra, Paola Cárdenas, Héctor Castellanos, Kateir Contreras, J. R. Castro

**Affiliations:** Units of Infectology and Dermatology, Department of Internal Medicine, National University of Colombia, 111321 Bogotá, Colombia

## Abstract

Toxic epidermal necrolysis is the most severe form of drug-induced skin reaction and includes denudation of >30% of total body surface area. The mechanism of disease is not completely understood, but immunologic mechanisms, cytotoxic reactions, and delayed hypersensitivity seem to be involved. We report a case of cephazolin-induced toxic epidermal necrolysis treated with intravenous immunoglobulin and N-acetylcysteine with excellent response.

## 1. Introduction

Drug-induced toxic epidermal necrolysis (TEN), also known as Lyell's syndrome, remains one of the most dramatic dermatological emergencies characterized by extensive destruction of the epidermis and mucosal epithelia that often can be caused by drugs [1]. TEN affects between 0.4 and 1.5 cases per million people every year [1, 2] with a mortality rate from 15% to 40%, with a large portion of patients dying from infections or multiorgan failure [3, 4].

The pathogenesis of drug-induced TEN is unknown, although several theories have been developed. Recent discoveries have shown that keratinocytes in TEN undergo apoptosis, not simply necrosis [5, 6]. Further research has elucidated that this apoptosis can be induced by interactions between cell surface death receptor Fas and its ligand, FasL or CD95L.

The management of these patients is primarily supportive, although use of corticosteroids and intravenous immunoglobulin (IVIG) therapy has been widely used with controversy.

We report a case of Cephazolin-induced toxic epidermal necrolysis with excellent response to N-acetylcysteine. Immunopathogenesis and current imputability of antibiotics in TEN will be discussed together with a review of the literature.

## 2. Case Report

A 38-year-old woman with a long history of type I diabetes was hospitalized in our institution for an acute episode of complicated urinary tract infection. She reported no history of allergy to antibiotics. Upon admission, physical examination revealed a temperature of 38°C, a pulse of 120 beats/min, and a blood pressure of 85/50. The patient developed septic and hypovolemic shock requiring aggressive fluid resuscitation and dopamine. Laboratory findings were white blood cells, 24 900/mm^3^ (85.8% segmented, 7.6% lymphocyte, 5.7% monocyte, 0.4% eosinophil, and 0.5% basophil); red blood cells, 435 × 10^4^/mm^3^; hemoglobin, 14.0 g/dL; platelets, 23.2 × 10^4^/mm^3^. Blood chemistry documented: glucose level, 305 mg/dL; blood urea nitrogen (BUN), 27 mg/dL; creatinine, 1.6 mg/dL; C-reactive protein, 70 mg/dL; IgG, 1050 mg/dL; IgA, 290 mg/dL; IgM, 132 mg/dL.

**Figure 1:**
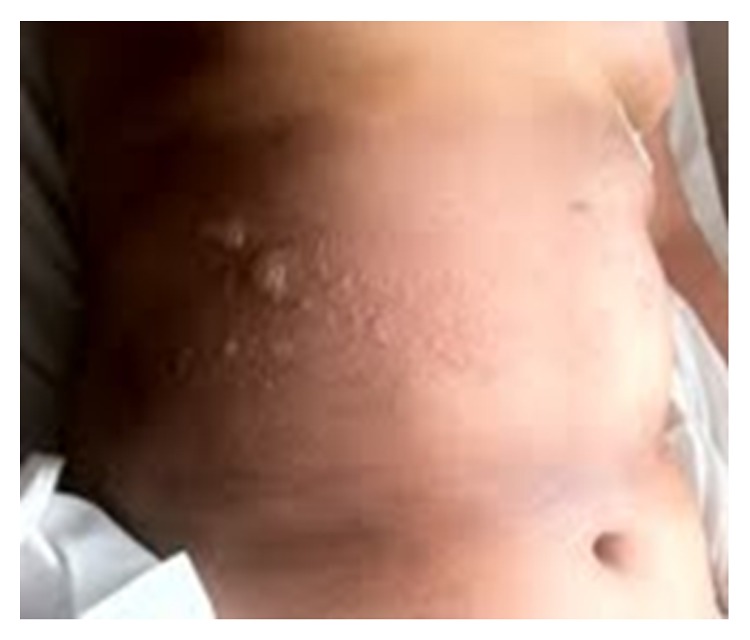


**Figure 2:**
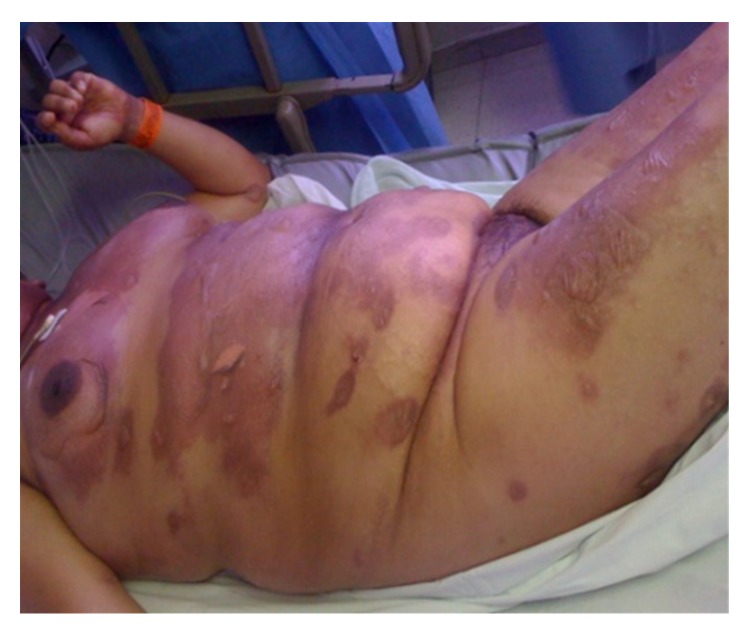


She was first administered cephazolin intravenously. Three days later, she developed an erythematous rash and flaccid blisters mainly on her back and arm (Figure 1). The administration of the antibiotic was stopped and was shifted to tigecycline according to the uroculture results. The cutaneous rash progressed onto the trunk, face, and limbs, reaching almost 52% of the body surface; blisters appeared progressively on the erythematous areas during the following 2 days (Figure 2).

**Figure 3:**
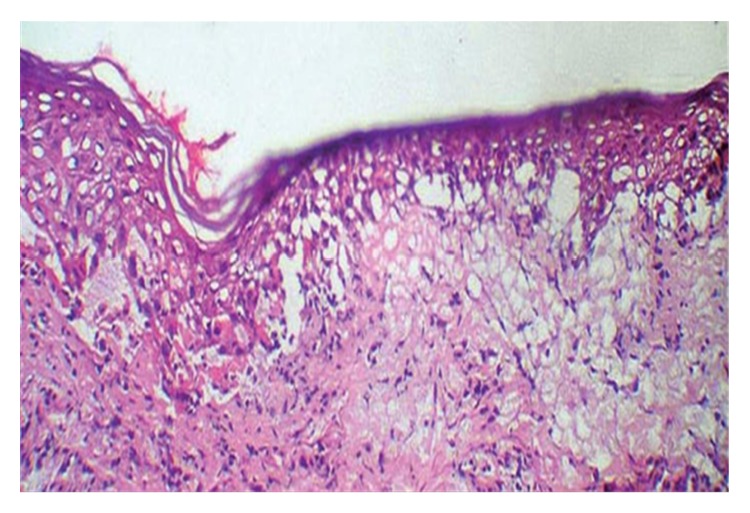


A calculated SCORTEN value of 3 predicted a 35.8% mortality rate. Bilateral conjunctivitis and erosions of the genital and oral mucosae also developed. The diagnosis of TEN was confirmed by a skin biopsy, showing a subepidermal blister with presence of rare mononuclear cells scattered between confluent necrotic keratinocytes (Figure 3).

The patient was placed on a fluidized bed and benefited from supportive and antiseptic measures, including daily baths. No systemic corticosteroids were given. Massive rehydration was undertaken. In addition, intravenous immunoglobulins were administered on a daily dosage of 1 g/kg body weight for three consecutive days. On completion of the immunoglobulin perfusions, the clinical examination revealed that progression to 75% BSA epidermal detachment occurred over the next 48 hours (Figure 4). The Nikolsky sign was positive. In the context of a septic patient we decided to administrate N-acetylcysteine 600 mg every 8 hours intravenously; two days later the erosive lesions of the skin and mucosae had dried and eyes were cured. She was discharged after 16 days, with complete reepithelialization.

## 3. Discussion

The Stevens-Johnson syndrome (SJS) and toxic epidermal necrolysis (TEN) represent different degrees of a severe, acute mucocutaneous reaction that often can be caused by drugs [1].

TEN is most commonly characterized by skin changes (scattered 2-ring target-like lesions with a dark-red center and lighter red halo and red macules with central blistering that can coalesce to larger areas of denuded skin), hemorrhagic mucositis (mouth, eyes, genitals, and respiratory tract), and systemic symptoms (fever, malaise, and possible internal organ involvement) [3].

SCORTEN, a TEN-specific severity of illness scale, has been proven to be an accurate predictor of mortality in patients with TEN by evaluating 7 independent risk factors (age, presence of malignancy, body surface area involved, serum urea nitrogen level, glucose level, bicarbonate level and heart rate) [7]. The main factors associated with TEN mortality are the occurrence of sepsis at the time of hospitalization (odds ratio [OR] 3.04), age (OR 1.11 per year of age), and total body surface area involved (OR 1.03 per percent of body surface area involved) [8].

In 74%–94% of cases, TEN is triggered either by preceding medication or by an infection of the upper respiratory tract. More than 100 different drugs are considered as having caused TEN. Among them, antibiotics represent one of the most common class of culprit drugs [2].

The prevalence of antibiotics being responsible for TEN ranges from 29% to 42% [2]. Almost all antibiotics have been implicated, but the beta-lactam compounds and the sulfonamide antibiotics exhibit the largest risk group. The relative risk of using aminopenicillins and cephalosporins (relative risk, 6.7 and 14, resp., IC 95%) is much lower than that of sulfonamide antibiotics (relative risk, 172 IC 95%) [9]. Cephalosporins that belong to the beta-lactam antibiotics group are indeed well-known inducers of TEN. However, cephazolin itself has never been reported to be responsible for TEN.

As sepsis is the most dreadful complication, antibiotics are commonly administered to TEN patients. The medication necessary to treat the TEN complications may be in some instances related to the drug that initiated the process, leading to the risk of cross-reactions. In our patient the treatment for urinary sepsis was responsible for the TEN leading to the risk of cross-reactions as well.

Recent discoveries in the immunopathogenesis of TEN have shown that keratinocytes undergo apoptosis, not simply necrosis, following different phases [10, 11]. Phase I is determined by the immunogenic impact of xenobiotics. It involves a lack of balance between activation and detoxification processes in keratinocytes. Phase II corresponds to early apoptosis. Generation of strongly electrophilic metabolites in TEN keratinocytes is thought to lead to disruption of the electron transfer chain in the mitochondria. In TEN keratinocytes it is likely that reactive oxygen species (ROS), acting as second messengers, increase gene transcription of the TNF-a and CD95 proapoptotic systems. [11] In late apoptosis (phase III) the proinflammatory cytokine TNF-a is believed to act as an autocrine/paracrine factor on neighbouring keratinocytes, thus spreading epidermal destruction. In phase IV, cells with ruptured mitochondria are at risk of death through a slow nonapoptotic mechanism resembling necrosis [12].

There is currently no specific treatment for TEN. Discontinuation of the suspected drug with supportive care (e.g., wound care, hydration, and nutritional support) forms the basis of treatment [4].

Intravenous immunoglobulin therapy (IVIG) is considered by many clinicians as a treatment option, blocking the binding of CD95L [13]. An updated review stratified results according to TEN and SJS, and 14 studies in patients with TEN were evaluated. The majority of studies reported positive results, while three cohort studies did not observe statistically significant improvement with IVIG administration [14]. In a subanalysis of these controlled trials, mortality rate for patients receiving IVIG was 27% compared with 30% for the predicted/control group. Because of the heterogeneity of the studies, a meta-analysis could not be conducted for IVIG in TEN or SJS.

N-acetylcysteine (NAC) is a cysteine derivative precursor of GSH. Abnormal inherited metabolic pathways are presumed in some cases, which could lead to a diminished detoxifying capacity. Administration of N-acetylcysteine enhances the oxidant buffering capacity of glutathione and inhibits nuclear factor kappa B, a transcription factor induced by tumor necrosis factor alpha and interleukin-6. Few patients have been successfully treated with high-dose intravenous NAC in open-label studies [15, 16]. Further studies are required to confirm the beneficial therapeutic effect of NAC in TEN.

Our patient was treated with IVIG with no response. A good response was achieved with N-acetylcysteine, showing the potential benefit of this drug in the TEN treatment.

Different studies had used thalidomide, cyclosporine, infliximab, azathioprine, methotrexate, cyclophosphamide, and plasmapheresis but were limited data to be recommended as first-line treatment [17, 18].

In conclusion, TEN is a complex pathology; although the incidence is relatively low, it is important to identify patients at risk to avoid delaying therapy. We suggest that several molecular targets that block different apoptosis/necrosis pathways should be attacked simultaneously in order to achieve optimal efficacy in the treatment of TEN. We consider that N-acetylcysteine is a good alternative to patients who did not respond to IVIG, especially in septic patients.

To the best of our knowledge, this is the first report of a cephazolin-induced toxic epidermal necrolysis.

## Figures and Tables

**Figure 4:**